# Autoactive Arabidopsis RPS4 alleles require partner protein RRS1-R

**DOI:** 10.1093/plphys/kiaa076

**Published:** 2020-12-18

**Authors:** Hailong Guo, Shanshan Wang, Jonathan D G Jones

**Affiliations:** The Sainsbury Laboratory, University of East Anglia, Norwich Research Park, Norwich NR4 7UH, UK

## Abstract

Autoactivity of an executor immune receptor due to mutations in putative ATP hydrolysis motifs requires the full-length allele of the cognate sensor immune receptor.

Dear Editor,

Plant disease resistance involves activation of defense upon detection of pathogen molecules by either cell-surface pattern recognition receptors (PRRs), leading to pattern-triggered immunity (PTI), or by intracellular nucleotide-binding (NB), leucine-rich repeat (NLR) immune receptors, leading to effector-triggered immunity (ETI). Pathogens suppress PTI by secreting effectors into host cells. These effectors can be recognized by NLRs, often encoded by Resistance (R) genes; the resulting ETI often culminates in the hypersensitive cell death response (HR; Jones and Dangl, 2006). NLR proteins have a similar architecture to animal NOD-like receptors, and directly or indirectly recognize pathogen effectors (van der Hoorn and Kamoun, 2008). Several plant NLRs function with another NLR protein. In these NLR pairs, one functionally specializes as sensor and the other as executor for effector perception and signaling initiation, respectively (Cesari et al., 2014). Resistance to* Ralstonia solanacearum* 1* (RRS1-R)* and Resistance to *Pseudomonas syringae* 4* (RPS4)* are a pair of Arabidopsis (*Arabidopsis thaliana*) NLR proteins that function together to recognize two bacterial effectors, PopP2, an acetyl-transferase from *Ralstonia solanacearum* and AvrRps4 from *Pseudomonas syringae* pv*. pisi*. The *RRS1-R* allele in Arabidopsis accessions Nd-1 and Ws-2 confers AvrRps4 and PopP2 recognition, whereas the Col-0 allele of *RRS1* (*RRS1-S*) confers only AvrRps4, but not PopP2, recognition (Le Roux et al., 2015; Sarris et al., 2015). Although AvrRps4 and PopP2 are recognized by the integrated WRKY domain in RRS1-R that mimics the effector’s authentic target, they derepress the executor NLR RPS4 by distinct mechanisms (Ma et al., 2018; Guo et al., 2020). Overexpressing wild-type *RPS4* reveals weak autoactivity that can be suppressed by RRS1-R (Wirthmueller et al., 2007; Huh et al., 2017). Previously defined autoactive alleles of RRS1/RPS4 result from mutations in the RRS1-R WRKY domain that mimic the outcome of effector action. In contrast to some NLRs (Tameling et al., 2006), less attention has been paid to *RPS4* autoactive alleles. We show here that in contrast to *RPS4* overexpression-dependent autoactivity, which is abolished by coexpression with RRS1-R, RRS1-R strongly enhances the HR triggered by *RPS4* autoactive alleles. These observations lead us to revise the view that RRS1-R acts solely as a negative regulator in suppressing RPS4-triggered autoactivity.

Several shared motifs in the NB-ARC domains contribute to NLR activation. Key among these (Supplemental Figure S1) are the P-loop (Walker-A motif), S in RNBS-A motif, hhhhDE (h represents a hydrophobic amino acid) of Walker B motif and conserved MHD motif (Meyers et al., 1999; Hanson and Whiteheart, 2005). Mutations in the P-loop motif of RPS4 (K242A) have been shown to abolish responsiveness to AvrRps4 and PopP2 (Williams et al., 2014). In other NLR proteins, certain mutations in the conserved motifs for ATP hydrolysis (RNBS-A and Walker-B motif) and weakened ADP-binding (MHD motif) can give autoactivation of the HR upon expression *in planta* (Tameling et al., 2006; van Ooijen et al., 2007). Sequence alignments reveal that amino acid S, D and D in the RNBS-A motif, the Walker B and MHD motif of RPS4 are highly conserved among plant NLRs (Supplemental Figure S1, Supplemental Materials and Methods). We postulated that amino acids S270, D315, and D509 in the NB-ARC domain of RPS4 might play a conserved role in regulating its activity, so we introduced S270F, D315E, and D509V mutations separately into RPS4 (Figure 1A, Supplemental Table S1).

**Figure 1:**
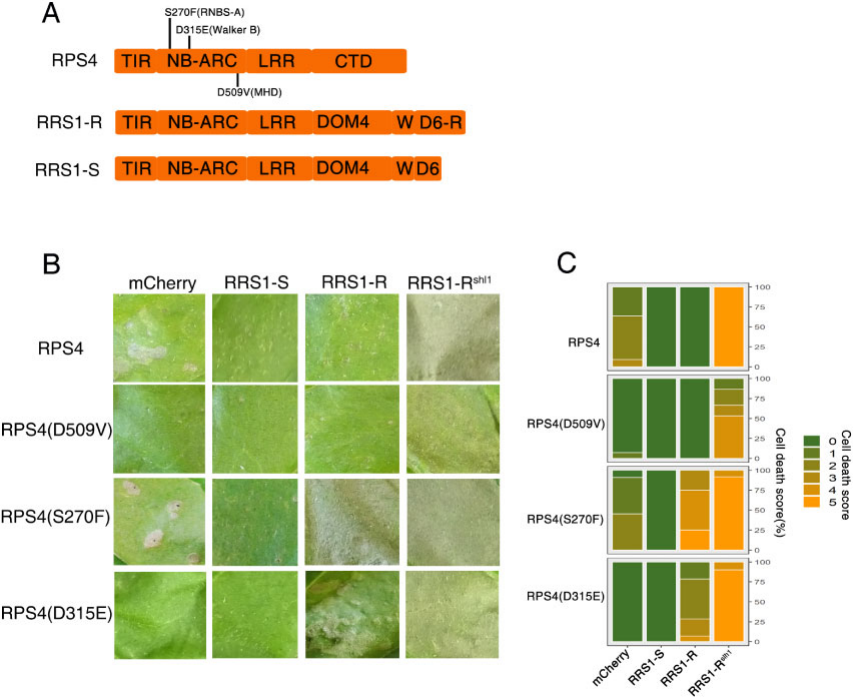
RRS1-R but nor RRS1-S facilitates the full activity of *RPS4* autoactive alleles. A, Schematic diagram of domain structures of RPS4 and RRS1-R/S. RPS4 consists of an N-terminal TIR, a central NB-ARC, LRR, and C-terminal domain (CTD). RRS1-R/S consists of TIR, NB-ARC, LRR, Domain 4 (DOM4), WRKY (W), and Domain 6 (D6-R/D6). Speciﬁc amino acids in the indicated motifs were subjected to mutational analysis. B, Assessing the autoactivity of RPS4 mutants in the presence and absence of RRS1-R/RRS1-S/RRS1-R^slh1^. Each tobacco leaf section was transiently inﬁltrated with indicated construct combinations. Leaves were photographed 5 d post-inﬁltration (dpi). C, Percentage representations of cell death scales in (B) at 5 dpi. Stacked bars are color coded to show the proportions (in percentage) of each cell death scale (0–5) out of total inﬁltrated leaves scored. A total of 11–14 leaves are scored for each stacked column.

Overexpression of RPS4 causes weak HR, and surprisingly, overexpression of RPS4(S270F) induces HR to a similar extent, but overexpression of RPS4(D315E) induces no HR (Figure 1B and C). Most strikingly, RPS4(D315E) exhibits HR when coexpressed with RRS1-R, but not RRS1-S (Figure 1B). Similarly, the weak HR caused by RPS4(S270F) is further enhanced by RRS1-R co-expression (Figure 1B and C), indicating that RRS1-R promotes defense activation by RPS4(D315E) and full activation of the RPS4(S270F). In contrast, RPS4 overexpression-mediated weak autoimmunity is suppressed by RRS1-R, indicating that RPS4(S270F) and RPS4(D315E) alleles act differently from wild-type RPS4. RPS4(S270F) and RPS4(D315E) probably represent an intermediate activation status, which can be further potentiated by RRS1-R. This potentiation is not explained by protein accumulation, because both RRS1-R and RRS1-S enhance RPS4 variant protein accumulation (Supplemental Figure S2A). Therefore, the extra C-terminal intrinsically disordered region (IDR) of RRS1-R must contribute to complex activation, since IDRs can facilitate protein oligomerization and activity (Faust et al., 2014). We did not detect autoactivation of RPS4(D509V) when overexpressed (Figure 1B). The RPS4(D509V) does not show autoactivity when coexpressed with RRS1-R, but RPS4(D509V) does show autoactivity in the presence of active RRS1-R^slh1^, albeit weakly (HR cell death is shown in Supplemental Figure S3), indicating that RPS4(D509V) behaves like wild-type RPS4 but with lower activity, most likely because of reduced protein accumulation (Supplemental Figure S2A).

RRS1-R is the only recessive NLR resistance gene; an RRS1-R/RRS1-S heterozygote loses RRS1-R function (Deslandes et al., 2002). We tested whether RRS1-S blocks the potentiation. Consistent with the recessive nature of RRS1-R, RRS1-S prevents the HR triggered by RRS1-R/RPS4(S270F) and RRS1-R/RPS4(D315E) (Figure 2A, Supplemental Figure S2B), consistent with the interfering action of a nonactivated form on an activated form (Sohn et al., 2014). The RRS1 “SH” motif in the TIR domain (S25 and H26), but not P-loop motif (K185), is indispensable for AvrRps4 or PopP2 recognition. Phosphorylation at Positions 1, 3, and 4 of the RRS1-R C-terminus is indispensable for PopP2, but dispensable for AvrRps4 recognition (Williams et al., 2014; Guo et al., 2020). We next investigated the role of the “SH” motif, P-loop motif, and C-terminal phosphorylation in RRS1-R-mediated potentiation. Agroinfiltration of RRS1-R variants carrying a mutated “SH” and P-loop motif did not induce HR when coexpressed with RPS4(S270F) or RPS4(D315E) (Figure 2B, Supplemental Figure S2C), respectively, indicating TIR-TIR domain heterodimerization and P-loop motif-dependent conformational changes are required for such potentiation. Similarly, agroinfiltration of RRS1-R^1/3/4A^ with RPS4(S270F) or RPS4(D315E) also fails to trigger HR (Figure 2B, Supplemental Figure S2C), again demonstrating the importance of C-terminal phosphorylation in RRS1-R function (Guo et al., 2020).

**Figure 2:**
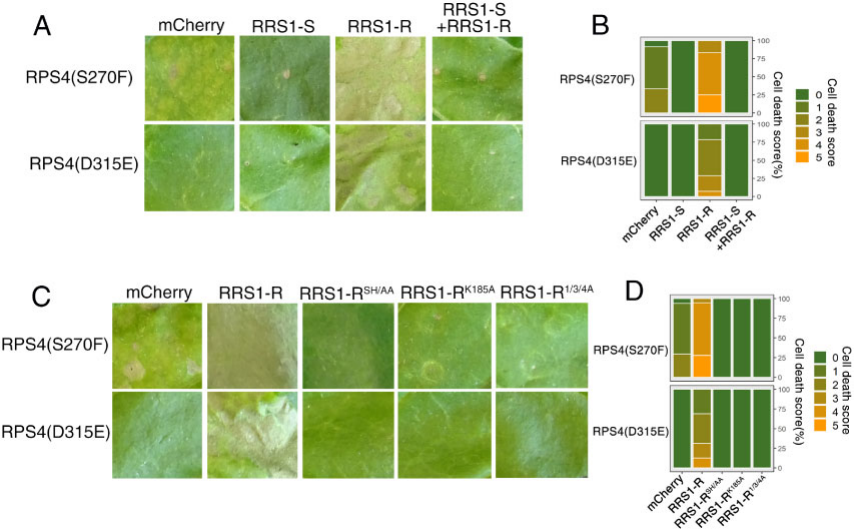
RRS1-R-mediated facilitation is recessive and requires its SH motif, P-loop, and C-terminal phosphorylation. A, Interference of RRS1-R-mediated HR potentiation by RRS1-S. Each tobacco leaf section was transiently inﬁltrated with indicated construct combinations. Leaves were photographed at 5 dpi. B, Percentage representations of cell death scales in (A) at 5 dpi. Stacked bars are color coded to show the proportions (in percentage) of each cell death scale (0–5) out of total inﬁltrated leaves scored. A total of 10–14 leaves are scored for each stacked column. C, RRS1-R-mediated HR potentiation requires its SH motif, P-loop, and C-terminal phosphorylation. Each tobacco leaf section was transiently inﬁltrated with indicated construct combinations. Leaves were photographed at 5 dpi. D, Percentage representations of cell death scales in (C) at 5 dpi. Stacked bars are color coded to show the proportions (in percentage) of each cell death scale (0–5) out of total inﬁltrated leaves scored. A total of 10–14 leaves are scored for each stacked column.

In summary, we report that in paired NLRs, full executor NLR autoactivity caused by mutations in proposed ATP hydrolysis motifs requires the cognate sensor NLR, showing sensor NLRs have a positive as well as negative function (Supplemental Figure S4). Future structural and biochemical investigations addressing these mutations are needed for a better mechanistic understanding of regulation and activation of the complex.

## Supplementary Material

kiaa076_Supplementary_Data

## Notes

Senior author. H.G. and J.D.G.J. designed the research. H.G. performed the experiments and analyzed data under the assistance of S.W. H.G. and J.D.G.J. wrote the paper. J.D.G.J. supervised the project and agrees to serve as the author responsible for contact and ensures communication.

The author responsible for distribution of materials integral to the findings presented in this article in accordance with the policy described in the Instructions for Authors (https://academic.oup.com/plphys) is: Jonathan D.G. Jones (jonathan.jones@tsl.ac.uk).
