# Supplementary material for: Autoactive Arabidopsis RPS4 alleles require partner protein RRS1-R
Source: Plant Physiol. 2020 Dec 18;185(3):761–4. doi: 10.1093/plphys/kiaa076 (PMC8133560; doi:10.1093/plphys/kiaa076)
Supplement: kiaa076_Supplementary_Data [file kiaa076_supplementary_data.zip › pp.01129.2020-s01.pdf]

Supplemental Material and Methods

Multiple sequence alignment

Protein amino acids sequences were retrieved from The Arabidopsis Information Resource (TAIR) and NCBI GenBank databases under the following accession numbers: *Ab*TN2, AT1G17615; *Ab*ZAR1, AT3G50950; *Ab*RPS4, AT5G45250; *Ab*RPS2, AT3G03600; *Ab*RPS5, AT1G12220; *Ab*ADR1, AT1G33560; *Ab*RPM1, AT3G07040; *Ab*NRG1a, AT5G66900; *Nb*NRC2, ALQ52761; *Nb*NRC3, QER78240; *Nb*NRC4, QER78241; *Nb*Roq1, ATD14363; *Sl*I-2, AAD27815;*St*Rx, CAB50786.

The sequence alignments were generate using online EMBL-EBI Clustal Omega (https://www.ebi.ac.uk/Tools/msa/clustalo/). The resulting alignment file was loaded in the Web service ESPript (http://espript.ibcp.fr/ESPript/ESPript/) (Robert and Gouet, 2014) to produce the alignment figure.

Plasmid construction

The C-terminally HF-tagged RPS4 variant constructs were generated using the Golden Gate assembly cloning procedure described previously (Ma et al., 2018). To generate RPS4 mutant, the fragments were amplified from wild-type RPS4 template using Goldern Gate cloning with primers containing BbsI site and the introduced mutations. All final constructs used in this study were validated by DNA sequencing. Primers sequences used for plasmid construction are shown in Table S1.

Agrobacterium-Mediated Tobacco Transient Assays (HR)

Agrobacterium-mediated transient expression were performed according to the method described previously in Guo et al. (2020). Briefly, *Agrobacterium* strain GV3101 harbouring indicated binary constructs were streaked on selective L-medium agar plates and grown for 48 h at 28°C. Bacteria were scraped from agar plates and then re-suspended in infiltration medium (10mM $MgCl_2$, 10mM MES, pH 5.6). For co-expression, each bacterial suspension carrying individual constructs was adjusted to OD_{600}=0.5 in the final mix for infiltration. The abaxial leaf sections of 5-week-old *Nicotiana. tabacum* fully-expanded leaves were infiltrated with 1 ml needleless syringe. HR phenotypes were photographed at 5 dpi.

Immunoblot analysis

Immunoblot analysis was performed according to the method described previously in Guo et al. (2020). Briefly, *Nicotiana benthamiana* leaves after transiently expressing the indicated constructs were ground in liquid nitrogen and then mixed with an equal volume of ice-cold GTEN buffer (10% glycerol, 150 mM Tris-HCl pH 7.5, 1 mM EDTA, 150 mM NaCl) supplemented with 10 mM DTT, 0.2% (vol/vol) Nodinet-40, Anti-protease tablet (Complete EDTA-free Roche) and 2% PVPP (Polyvinylpolypyrrolidone). The lysates were then centrifuged at 5000 g for 30 min at 4°C, and the supernatant was filtered through 0.45 mm filters. The filtered protein extract mixed with 3xSDS loading buffer (30% glycerol, 3.3% SDS, 94 mM Tris-HCl pH 6.8, 0.05% (vol/vol) bromophenol blue) supplemented with 10 mM DTT were separated by SDS-PAGE. Proteins were transferred to Immobilon-P PVDF membranes (Merck Millipore) and incubated with Horseradish Peroxidate (HRP) conjugated antibodies (Anti-FLAG M2, 1:10000 dilution). Chemiluminescence detection was carried out by incubating the membrane with developing reagents (SuperSignal West Pico & West Femto) and followed by using ImageQuant LAS 4000 (Life Sciences). For quantification, the relative intensities of immunoblotting band were processed and quantified with ImageJ software (National Institutes of Health).

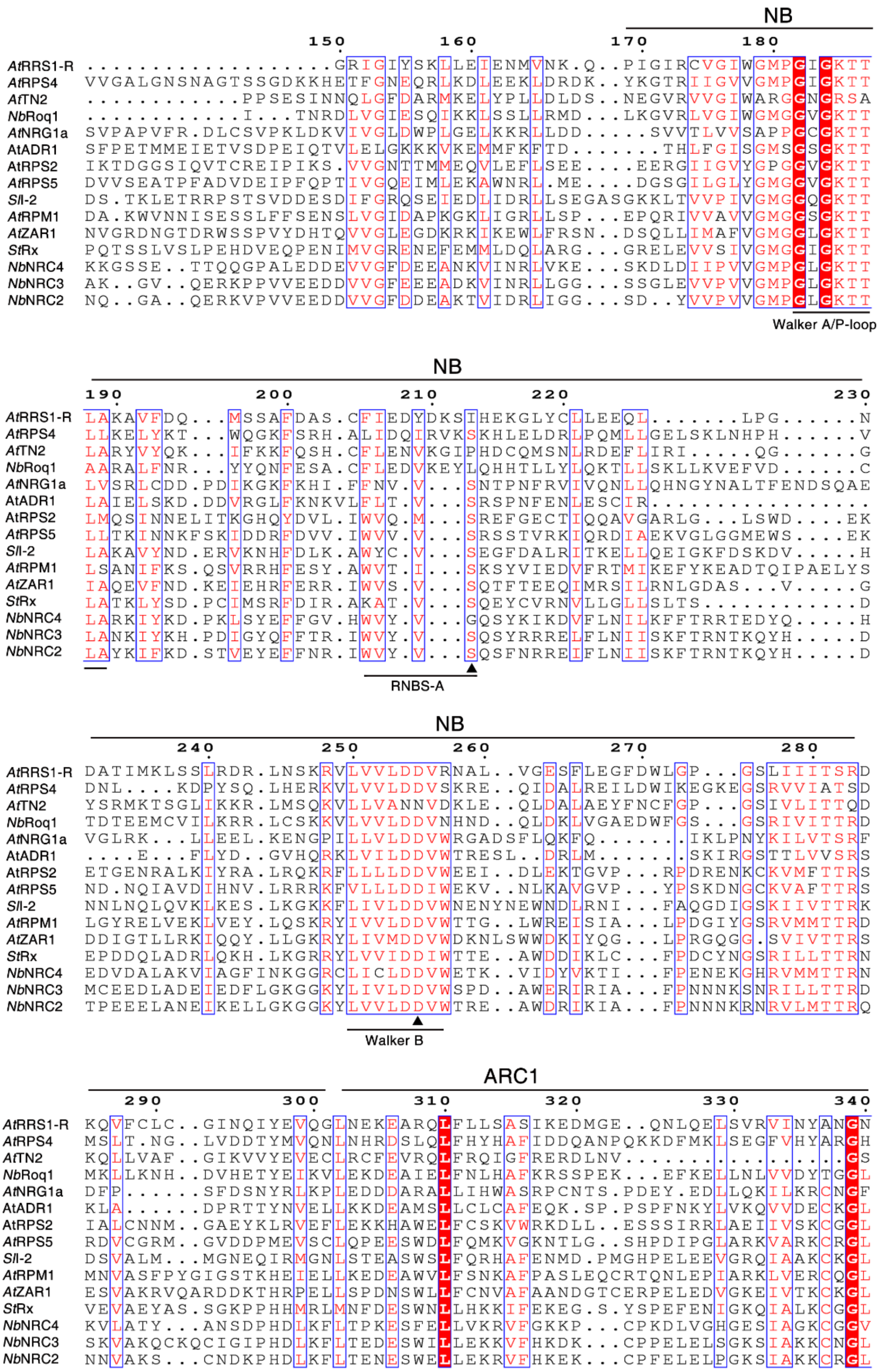
NB
150
160
170
180
AtRRS1-R
AtRPS4
AtTN2
NbRoq1
AtNRG1a
AtADR1
AtRPS2
AtRPS5
SlI-2
AtRPM1
AtZAR1
StRx
NbNRC4
NbNRC3
NbNRC2
Walker A/P-loop
NB
190
200
210
220
230
RNBS-A
NB
240
250
260
270
280
Walker B
ARC1
290
300
310
320
330
340

```
          ARC1                                  ARC2
                350       360       370            380       390
AtRRS1-R  PLAINVYGRELKGK.KKLSEMETAFLKLKRRPP......FKIVDAFKSTYDTLSDNE.KN
AtRPS4    PLALKVLGGELNK..KSMDHWNSKMKKLAQSPS......PNIVSVFQVSYDELTTAQ.KD
AtTN2     ELSSPISGTESS................................................
NbRoq1    PLALKVLGSLLYK..EDLDVWISTIDRLKDNPE......GEIMATLKISFDGLRDYE.KS
AtNRG1a   PIVIEVVGVSLKGRS..LNTWKGQVESWSEGEKILGKPYPTVLECLQPSFDALDPNL.KE
AtADR1    PLSLKVLGASLKNKP..ERYWEGVVKRLLRGEAADETHESRVFAHMEESLENLDPKI.RD
AtRPS2    PLALITLGGAMAHR.ETEEEWIHASEVLTRFPAEMKGMN.YVFALLKFSYDNLESDLLRS
AtRPS5    PLALNVIGEAMACK.RTVHEWCHAIDVLTSSAIDFSGMEDEILHVLKYSYDNLNGELMKS
Sl-2      PLALKTLAGMLRSKSEV.EEWKRILRSEIWELPH.....NDILPALMLSYNDLPAHL.KR
AtRPM1    PLAIASLGSMMSTK.KFESEWKKVYSTLNWELNN.NHELKIVRSIMFLSFNDLPYPL.KR
AtZAR1    PLTIKAVGGLLLCKDHVYHEWRRIAEHFQDELRGNTSETDNVMSSLQLSYDELPSHL.KS
StRx      PLAITVIAGLLSKMGQRLDEWQRIGENVSSVVS..TDPEAQCMRVLALSYHHLPSHL.KP
NbNRC4    PLAVVVIAGALRGRPNT.SDWIRVERNVVQHLYT.NS.EESCLKFVEMSYDHLPQEV.QT
NbNRC3    PLAIVVIAGALIGKGKTSREWKQVDESVGEHLIN.KDQPENCNKLVQLSYDRLSYDL.KA
NbNRC2    PLAIVVIAGALIGKGKTTREWELVADSVGEHLIN.RD.PENCKKLVQMSYDRLPYDL.KA
```

```
                              ARC2
             400       410       420                 430
AtRRS1-R  IFLDIACFFQGENVNYVIQLLEGC...GFFPHVE............IDVLVDKCLVTISE
AtRPS4    AFLDIACFRSQDKDY.VESLLASSDLGSAEAMSA...........VKSLTDKFLINTCD
AtTN2     ............................................................
NbRoq1    IFLDIACFFRGYNQRDMTALFHAS...GFHPVLG............VKTLVEKSLIFILE
AtNRG1a   CFLDMGSFLEDQKIR.ASVIIDMWVEL......YGK..GSSILYMYLEDLASQNLLKLVP
AtADR1    CFLDMGAFPEDKKIP.LDLLTSVWVER......HDI..DEETAFSFVLRLADKNLLTIVN
AtRPS2    CFLYCALFPEEHSIE.IEQLVEYWVGEGFLTSSHGVN.TIYKGYFLIGDLKAACLLE.TG
AtRPS5    CFLYCSLFPEDYLID.KEGLVDYWISEGFINEKEGRERNINQGYEIIGTLVRACLLLEEE
Sl-2      CFSFCAIFPKDYPFR.KEQVIHLWIANGLVPVKDEI..NQDLGNQYFLELRSRSLFEKVP
AtRPM1    CFLYCSLFPVNYRMK.RKRLIRMWMAQRFVEPIRGVK.AEEVADSYLNELVYRNMLQVIL
AtZAR1    CILTLSLYPEDCVIP.KQQLVHGWIGEGFVMWRNGRS.ATESGEDCFSGLTNRCLIEVVD
StRx      CFLYFAIFTEDEQIS.VNELVELWPVEGFLNEEEGKS.IEEVATTCINELIDRSLIFIHN
NbNRC4    CFLYCGVFPRGFDIP.SWKVIRLWIAEGLIKPQESYT.LEEIAEFYINDLVNRNLVILQQ
NbNRC3    CFLYCGAFPGGFEIP.AWKLIRLWIAEGFIQYKGHLS.LECKAEDNLNDLINRNLVMVMQ
NbNRC2    CFLYCGAFPGGSEIS.AQKLICLWIAEGFIQYQGPLT.LEDIAEDHLNDLVNRNLVMVMK
```

```
                              ARC2
                         440       450       460       470       480
AtRRS1-R  NR.............VWLHKLTQDIGREIINGETVQ.IERRRRLWEPWSIKYLLEYNEHK
AtRPS4    GR.............VEMHDLLYKFSREVDLKASNQDGSRQRRLWLHQHIIKG.......
AtTN2     ............................................................
NbRoq1    DK.............IQMHDLMQEMGRQIAVQESP.....MRRIYRPEDVKDA.......
AtNRG1a   LGTNEHEDGFYNDFLVTQHDILRELAICQSEFKEN.........................
AtADR1    NPRFGDVHIGYYDVFVTQHDVLRDLALHMSNRVDV.........................
AtRPS2    DE..........KTQVKMHNVVRSFALWMASEQGT.........................
AtRPS5    RN..........KSNVKMHDVVREMALWISSDLGK.........................
Sl-2      NPSKRNI.....EELFLMHDLVNDLAQLASSKLCIRL..........EESQGSHM.....
AtRPM1    WNPFGR......PKAFKMHDVIWEIALSVSKLE.RFC..........DVYNDDSD.....
AtZAR1    KTYSGT......IITCKIHDMVRDLVIDIAKKDS.FS..........NP.....E.....
StRx      FSFRGT......IESCGMHDVTRELCLREARNM.NFV..........NVIRGKSD.....
NbNRC4    KRSDGQ......IKTCRLHDMLHQFCKKEAS.NKWLF..........QEVSLTPD.....
NbNRC3    RTSDGQ......IKTCRLHDMLHEFCRQEAMKEENLF..........QEIKLGAE.....
NbNRC2    RSSSGQ......IKTCRVHDMLHEFCRHEAMMEENLF..........QEIKRGQE.....
                             MHD
```

```
                                   ARC2
              490       500          510             520       530
AtRRS1-R  ANGEPKTTFKRAQGSEE...IEGLFLDTSN......LRFDLQPSAFKNMLNLRLL..KIY
AtRPS4    ...GIINVLQNKMKAAN...VRGIFLDLSEVEDETSLDR....DHFINMGNLR..YLKFY
AtTN2     ............................................................
NbRoq1    ........CIGDMRKEA...IEGLLLTEPEQFEEGELEYMYSAEALKKTRRLRILVKEYY
AtNRG1a   .................LERKRLNLEILE.N....T....FP.....DWCL.........
AtADR1    .................NRRERLLMPKTE.P....V....LP.....REWEKN.......
AtRPS2    ...................YKELILVEPS....MGHTEAPKAENWRQALVIS.......
AtRPS5    ...................QKEKCIVRAG....VGLREVPKVKDWNTVRKIS.......
Sl-2      .....LEQCRHLSYSIGFNGE............FKKLTPLYKLEQLRTLLPIR.......
AtRPM1    .....GDDAAETM..EN.YGSRHLCIQKEMTPDSI......RATNLHSLLVCS.......
AtZAR1    .....GLNCRHLGISGNFDE.KQIKVNH.KLRGVVSTTK..TGEVNKL...........
StRx      .....QNSCAQSM.QRSFKSRSRIRIHKVEE................LAWCRN.......
NbNRC4    .....QAI...PI.EDP.NKSRRLCIQPSNLKDFLSKKP..SAEHVRSFYCFS.......
NbNRC3    .....QYF...PG.KRELATYRRLCIHS.SVLEFISTKP..SGEHVRSFLSFS.......
NbNRC2    .....HSF...PE.KQELASYRRLCIHS.SVSEFLSTKP..FAEHVRSFLCFA.......
```

Supplemental Figure S1. Multiple sequence alignment of NB-ARC domains of multiple NLRs.

The amino acid sequence of RPS4 was aligned with representative NLRs from different classes. The subdomain and motifs are annotated as black lines above and below the aligned sequences, respectively. The three conserved amino acid residues for mutation experiments are marked with black triangles. (For details on the alignment, see “Supplemental Materials and Methods.”)

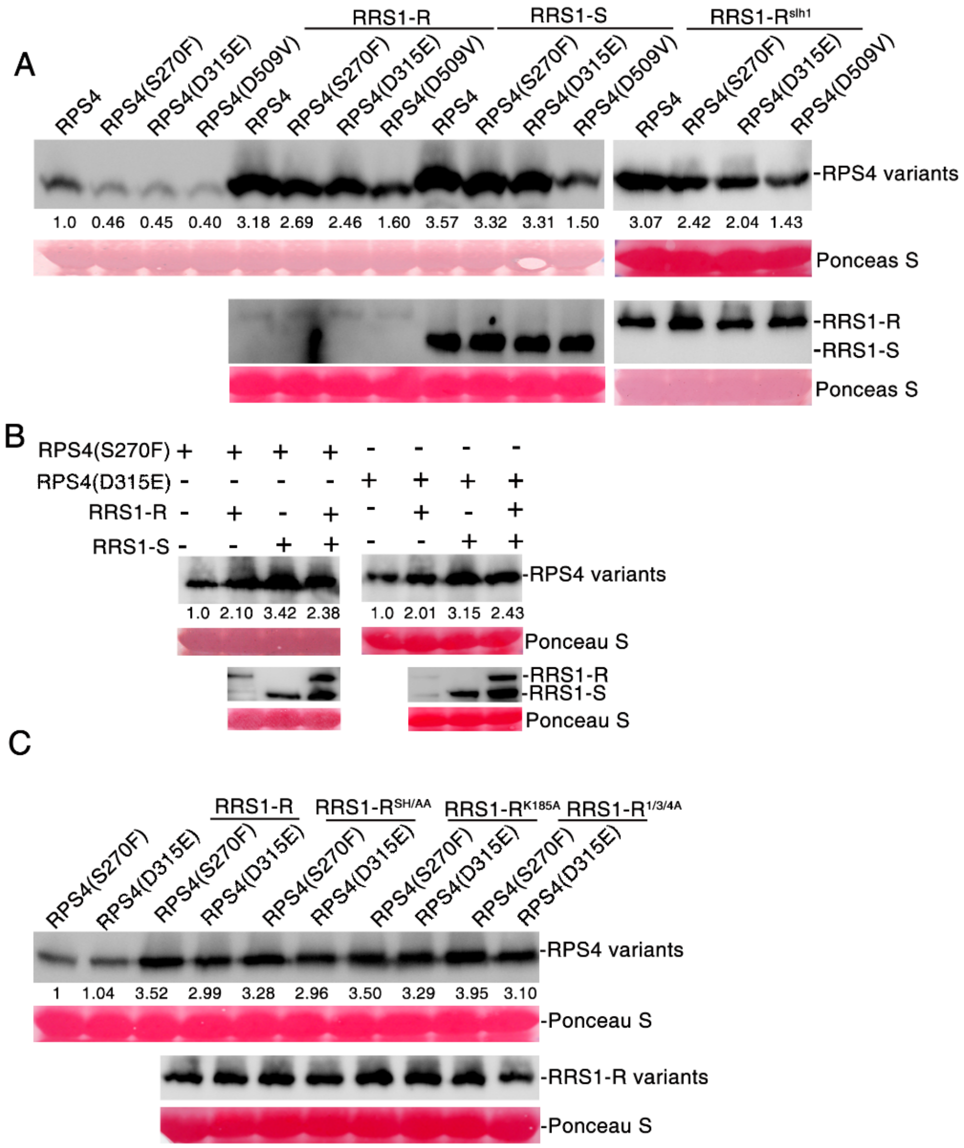

Supplemental Figure S2. Expression levels of wild-type and mutant RPS4.

(A-C) *Nicotiana benthamiana* leaves were agroinfiltrated with indicated constructs to express RPS4 and RRS1-R/S variants. RPS4 variants and RRS1-R/S variants are tagged with HF and GFP and detected by immunoblot assay using anti-FLAG and anti-GFP antibody, respectively. Ponceau S staining of Rubisco served as a loading control. RPS4 variants protein bands shown in western blots were quantified by ImageJ.

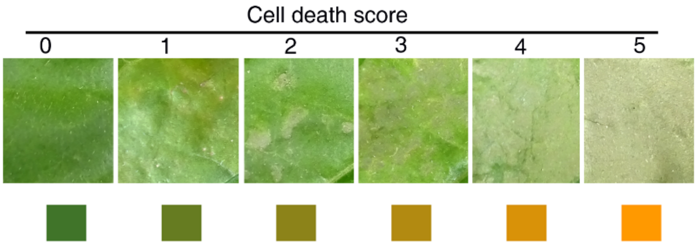

Supplemental Figure S3. Representative images of cell death score.

Representative images of the 0–5 scale used to determine the extent of cell death at 5 dpi. 0, no cell death; 5, full cell death.

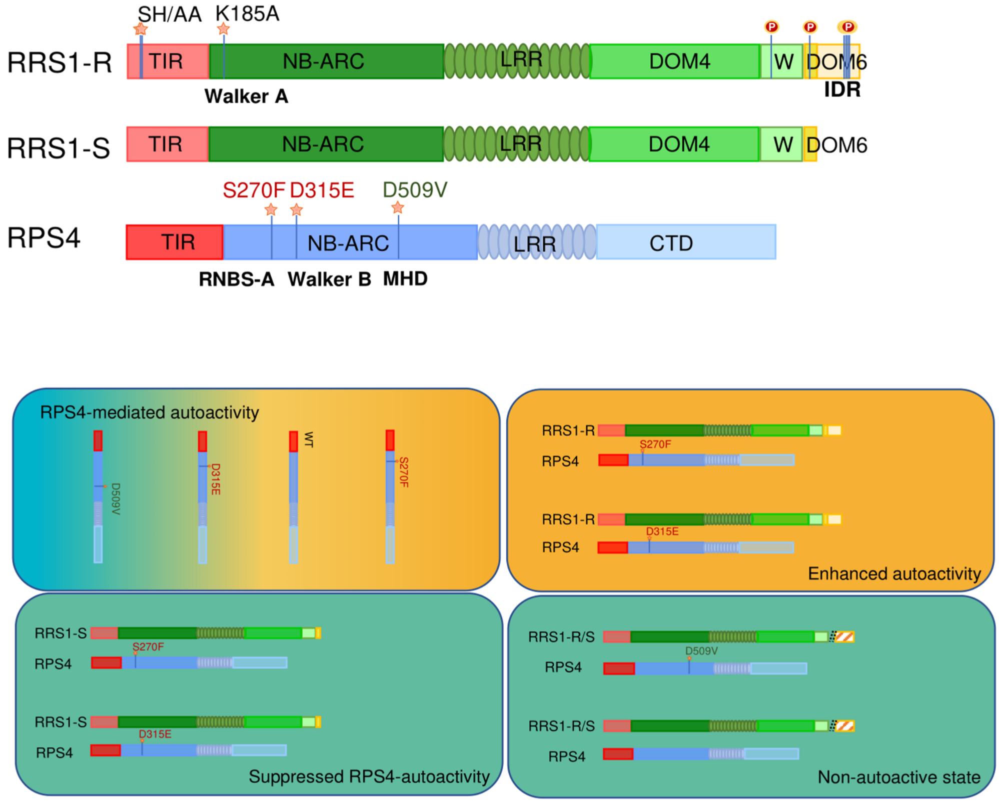

Supplemental Figure S4. A model illustrating the potentiation of RPS4 autoactive alleles by RRS1-R but not RRS1-S.

The relative activation potential of each RPS4 allele is indicated from cyan (non-autoactive) to yellow (autoactive). The depth of the color represents the (in)activation strength.

Supplementary Table 1. Primers used for plasmid construction in this study.

Purpose	Gene/ Fragments	Vector	Name	Sequence (5’-3’)
Generation of mutatants	RPS4(S270F)	pICSL 01005	S270F-F	AATGAAGACATTTTAAGCACTTGGAGTTGGATCGCT
	RPS4(S270F)		S270F-R	AATGAAGACATTAAACTTTACACGGATTTGATCGAT
	RPS4(D315E)		D315E-F	AATGAAGACATGAAGTTAGTAAAAGGGAACAAATAG
	RPS4(D315E)		D315E-R	AATGAAGACATCTTCATCAAGTACAACAAGCACTTT
	RPS4(D509V)		D509V-F	AATGAAGACATGTCCTATTATATAAATTTTCAAGGG
	RPS4(D509V)		D509V-R	AATGAAGACATGGACATGCATCTCCACTCGGCCATC
