# Supplementary figures and images for: Autoactive Arabidopsis RPS4 alleles require partner protein RRS1-R
Source: Plant Physiol. 2020 Dec 18;185(3):761–4. doi: 10.1093/plphys/kiaa076 (PMC8133560; doi:10.1093/plphys/kiaa076)

Fig S4

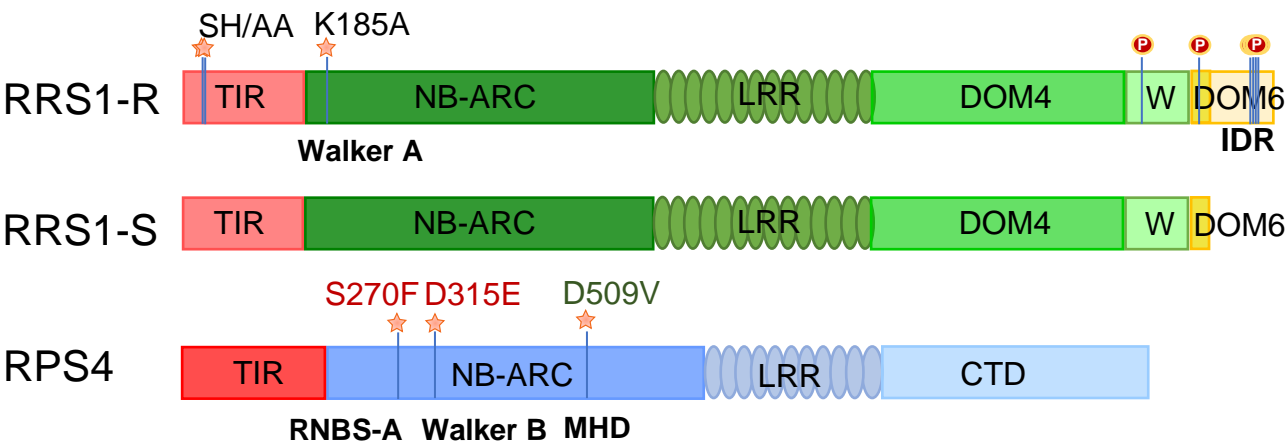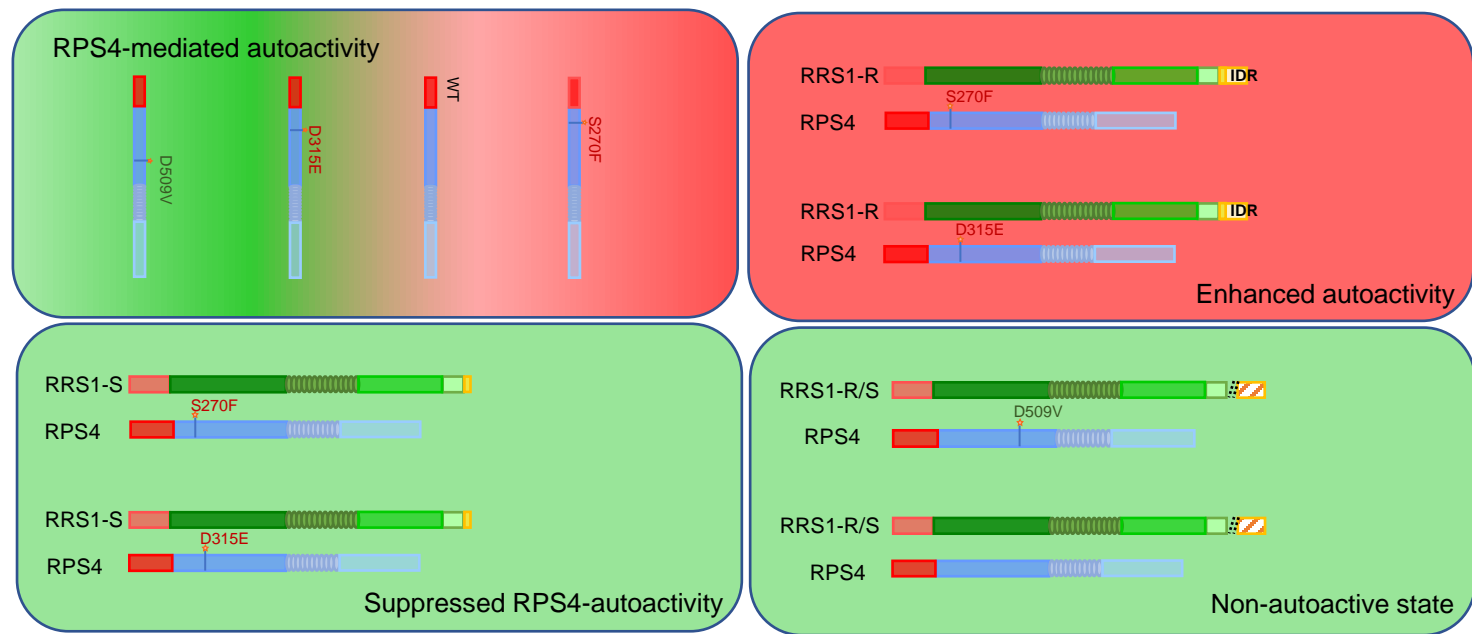

Supplement: kiaa076_Supplementary_Data [file kiaa076_supplementary_data.zip › pp.01129.2020-s02.pdf]
